# Knowledge of medications for opioid use disorder and associated stigma among primary care professionals

**DOI:** 10.1080/07853890.2024.2399316

**Published:** 2024-09-05

**Authors:** Hannah Piscalko, Lindsay Y. Dhanani, Daniel Brook, O. Trent Hall, William C. Miller, Vivian Go, Janet E. Simon, Berkeley Franz

**Affiliations:** a College of Public Health, Ohio State University, Columbus, OH; b School of Management and Labor Relations, Rutgers University, Piscataway, NJ; c Department of Psychiatry and Behavioral Health, Wexner Medical Center, Ohio State University, Columbus, OH; d Gillings School of Global Public Health, University of North Carolina Chapel Hill, Chapel Hill, NC; e College of Health Sciences and Professions, Ohio University, Athens, OH; f Heritage College of Osteopathic Medicine, Ohio University, Athens, OH; g Institute to Advance Health Equity, Ohio University, Athens, OH

**Keywords:** Opioid-related disorders, buprenorphine, training, primary care, methadone, addiction

## Abstract

**Background:**

Medications for opioid use disorder (MOUD) are the gold standard. However, significant barriers limit their use in the primary care setting, including limited knowledge of the medications and stigmatizing attitudes. In this study, we assess knowledge levels among primary care-aligned professionals (PCPs) currently in practice, and whether knowledge of MOUD is associated with stigma and treatment attitudes.

**Participants and methods:**

Using rosters from the state of Ohio licensing boards, we surveyed 403 physicians, nurse practitioners, and physician associates in 2022, on the mechanism of different MOUD, as well as stigma and treatment attitudes. To assess MOUD knowledge, we employed descriptive and bivariate statistics. We fit four linear regression models, which controlled for empathy towards patients with OUD and provider demographics to assess the relationship between MOUD knowledge and four endpoints: stigma, perceived controllability of opioid use, perceived vulnerability to opioid use disorder, and support for abstinence-only treatment.

**Results:**

43% of participants correctly identified the mechanism of all 3 medications whereas 13% of participants did not identify the mechanism of any MOUD correctly. MOUD knowledge was higher among physicians as compared to nurse practitioners and physician associates. Lower MOUD knowledge was associated with more negative attitudes towards patients with OUD and MOUD treatment.

**Conclusion:**

Expanding access to MOUD treatment requires a trained and willing health-care professional (HCP) workforce. Our findings highlight considerable variation in clinician knowledge of MOUD and suggest that knowledge levels are also related to negative attitudes towards patients with OUD and MOUD. Training interventions that increase knowledge, as well as focus on stigma reduction, are critical for reducing the longstanding treatment gap for opioid use disorder.

## Introduction

In 2017, the Department of Health and Human Services declared that the opioid epidemic was a public health emergency, underscoring the tremendous morbidity and loss of life associated with opioid use [1]. Overdoses have increased more than five-fold since 1999 [2], and remain a leading cause of injury-related death in the US [3]. Beyond overdose, injection opioid use has contributed to growing rates of HIV, hepatitis C virus infection, endocarditis, and skin and soft tissue infections. These infectious complications have exacted a substantial toll on the health care system [4].

Treatment for OUD is effective and reduces overdose risk, decreases infectious disease transmission, and improves social and economic outcomes [5]. Medications for opioid use disorder (MOUD), including buprenorphine, methadone, and naltrexone, are considered the gold standard in treatment [6], each with advantages for different patient populations. MOUD reduce opioid use and help retain patients in treatment; but only 1 out of 10 patients with OUD successfully accesses MOUD [7]. Overall, the estimated gap between treatment need and capacity in the US is between 1 and 1.4 million people [8].

Factors at the patient, clinician, and system levels interact to foster the underuse of MOUD [9]. Longstanding regulation of buprenorphine and methadone has contributed to significant stigma among both patients and health-care professionals. One of the most intractable challenges with increasing MOUD access is the availability of health-care professionals (HCPs) who are willing to prescribe MOUD, especially clinicians in primary care-aligned fields [10–13]. Because training in addiction medicine remains variable and generally limited in health professions education [14,15], many providers do not have the knowledge, experience, and confidence to effectively screen and diagnose opioid use disorder, and initiate MOUD [16–18]. Because of these barriers, many patients with OUD remain underserved and unable to access evidence-based treatment.

In qualitative studies, a lack of knowledge about OUD treatment is commonly cited as a barrier to MOUD prescribing among HCPs, but the extent of OUD knowledge among existing health care providers has rarely been examined quantitatively and whether limited knowledge is associated with negative attitudes has not been fully examined in the context of MOUD. For example, limited education may contribute to stereotypes about OUD and MOUD, including beliefs that OUD is a simply a bad choice and that MOUD substitutes one drug for another which may limit willingness to prescribe MOUD [19–21].

Increasing knowledge is among the most common strategies tested for improving negative attitudes towards stigmatized groups [22,23]. The theorized mechanism for changing attitudes through educational interventions is that with greater knowledge, individuals will rely less on negative stereotypes when forming attitudes about a stigmatized group or treatment [24]. Much of the literature on knowledge and stigma comes from mental health research which has demonstrated that improving knowledge on mental health conditions, through educational initiatives such as Mental Health First Aid, have been associated with more positive attitudes towards individuals living with stigmatized psychiatric conditions [25,26]. Similarly, efforts to frame addiction as a chronic, relapsing brain disease and convey that effective treatments exist, have been prioritized within health communication campaigns to reduce stereotypes about the controllability of substance use disorders (SUDs) and stigma toward patients [27–31].

It is thus plausible that improving knowledge on the physiology of opioid use disorder and the mechanisms of MOUD may decrease negative attitudes towards this patient population and towards MOUD by addressing and reducing reliance on stereotypes. Systematic reviews of the literature have found conflicting evidence, however, regarding whether increasing knowledge about SUDs may also improve attitudes towards patients with OUD and increase support for evidence-based treatment [23,32]. Additional research is needed, specifically in the context of OUD, to understand whether improved knowledge is associated with more positive attitudes. In this study, we aim to address this gap by examining knowledge among currently practicing primary care-aligned providers working in Ohio, and identifying attitudes associated with MOUD knowledge that may be targeted in future interventions to increase MOUD prescribing.

## Methods

### Study population

In late 2022, we surveyed 403 health-care professionals currently licensed to practice in Ohio. This study is part of a parent study for which we are developing a buprenorphine prescribing support program for rural primary care professionals in Ohio. We chose to focus on Ohio because it is a state heavily impacted by the opioid epidemic [33] and because it is a state with additional statewide regulation of buprenorphine prescribing [34]. Although there are limitations to a single-state approach, we elected this design to assess urban–rural differences in prescribing within a single state context. The statewide survey was the first aim of the parent study and eligibility criteria included being an HCP eligible to prescribe medications such as MOUD, which, in Ohio, includes physicians, nurse practitioners (NPs) and physician associates (PAs). Because we were most interested in prescribing in the primary care setting, we included family and internal medicine providers, as well as HCPs from closely aligned disciplines who were likely to interact with patients with OUD, including: obstetrics/gynecology, emergency medicine, addiction medicine, infectious disease, pain medicine, and psychiatry.

Using G*power, we conducted an a priori power analysis resulting in a goal of 400 participants to adequately power our regression analyses predicting our primary prescribing outcomes and secondary attitudinal outcomes. To increase participation and diversity in our sample, we recruited through multiple channels: 1) we emailed 20,143 random HCPs, using contact information available in the State Board of Medical and Nursing Licensing rosters; 2) we placed public advertisements for participation in medical professional society newsletters and listservs in Ohio; and 3) we partnered with health professions training programs in Ohio to email the survey invitation to alumni.

We closed the survey as soon as possible after reaching our a priori goal which resulted in 403 complete responses. Incomplete responses were removed using listwise deletion. Because we do not know the total number of invitations sent, it is not possible to calculate a response rate. Even among the known email invitations sent to the HCPs in the state medical licensing board roster, the percentage of emails opened ranged from 31.2% to 60.2% with each send. We sent two reminder emails approximately 3 and 7 days after the original email invitation. After sending out invitations in batches, our survey was in the field for approximately 2.5 months, closing in late December 2022. A total of 659 participants began the survey and 403 completed all items (62% completion rate). We provided a $20 Amazon gift card to all participants completing the survey. To maintain anonymity, participants were redirected to a second survey to provide an email address to which to send the gift card. The study was approved by the Ohio University internal review board (22-X-74), and all respondents provided electronic informed consent prior to participation. The study adhered to the principles of the Declaration of Helsinki.

### Data and measures

The survey contained primarily closed-ended questions on the mechanisms of different MOUD, attitudes towards patients with OUD and MOUD treatment, as well as demographic characteristics. We had five primary outcomes. The first outcome was correctly identifying the pharmacological mechanism of action for three common MOUD: methadone, buprenorphine, and naltrexone. We measured these using 4-item multiple-choice questions with one correct answer and three distractors. Participants indicated whether each option was true or false. We also created a composite variable measuring the percentage of the three MOUD that were correctly identified.

We next explored whether our first primary outcome, MOUD knowledge, predicted four additional outcomes: attitudes towards patients with OUD, including explicit bias or stigmatized attitudes toward patients with OUD; belief that patients are responsible for their addiction; belief among HCPs that they are not vulnerable to addiction; and support for recovery without medication. Stigmatized attitudes were measured using a previously validated 8-item measure [35], with five response options ranging from (1) strongly disagree to (5) strongly agree, which showed strong internal consistency using Cronbach’s alpha (α = 0.80). Controllability was measured using a four-item scale of beliefs about whether patients are in control of or are responsible for their addiction, with five response options ranging from (1) strongly disagree to (5) strongly agree (α = 0.72). The scale was previously adapted for a study on opioid use disorder, and was originally used to measure beliefs about control for people who inject drugs [36,37]. The vulnerability item measured HCP beliefs about their own vulnerability to opioid use disorder (α = 0.80), with five response options ranging from (1) strongly disagree to (5) strongly agree [36]. Because fostering empathy towards patients with OUD is often the focus of education on OUD, we also controlled for empathy to help isolate the relationship between knowledge about the mechanisms of MOUD and negative attitudes. We measured empathy using a previously validated scale of empathy towards patients with OUD [38]. This six-item measure (α = 0.93) asked participants to indicate the extent to which they felt various responses toward patients with OUD (e.g. sympathetic, warm), on a five-point response scale ranging from (1) not at all to (5) extremely. Demographic items included sex, age, training credentials, area of practice, rural/urban location [39], previous receipt of the X-waiver, and the average number of hours worked per week.

### Analysis

To assess the first endpoint in our sample, MOUD knowledge, we employed descriptive and bivariate statistics to characterize the percent of HCPs able to correctly identify each MOUD mechanism of action, by both provider type and area of practice. For our remaining four endpoints, we assessed the impact of HCP prescribing knowledge on attitudes towards patients with OUD and toward MOUD. We fit four linear regression models for each primary outcome, controlling for empathy towards patients with OUD and provider demographics. These models identify the expected change in each primary outcome when knowledge changes by one unit, holding all other variables constant [40]. All statistical analyses were conducted using Stata 15 [41].

## Results

### Descriptive and bivariate analyses

An examination of provider demographics indicated that 30% (*n* = 121) of participants were NPs; 43% (*n* = 174) worked as physicians; and 27% (*n* = 109) were PAs (Table 1). Approximately 61% (*n* = 245) were female; 82% (*n* = 330) were White; and the average age of respondents was 42.3 years old (SD = 12.0). Participants worked an average of 41.3 h per week (SD = 13.1). Further, 54% (*n* = 285) of participants worked in family medicine, with the remaining in other primary care-aligned disciplines.

**Table 1.** Descriptive statistics for study variables.

			Scale or range	
Variables	Mean	SD	Min	Max
Knowledge of all 3 medications	0.43	0.50	0	1
Buprenorphine knowledge	0.60	0.49	0	1
Methadone knowledge	0.67	0.47	0	1
Naltrexone knowledge	0.64	0.48	0	1
Bias/stigma	2.04	0.70	1	5
Controllability	2.29	0.74	1	5
Vulnerability	3.43	0.84	1	5
Abstinence-based recovery	2.58	1.03	1	5
Rural location	0.21	0.41	0	1
Years in job	17.78	11.57	1	35
Work hours	41.31	13.14	0	100
Female	0.61	0.49	0	1
Age (M ± SD)	42.43	11.96	25	75
Physician assistant	0.27	0.44	0	1
Nurse practitioner	0.30	0.46	0	1
Physician	0.43	0.50	0	1
Receipt of X-waiver	0.48	0.50	0	1

SD: standard deviation; Min: minimal value; Max: maximum value.

Approximately 67% (*n* = 285) of participants correctly identified the mechanism of methadone, which was the MOUD most often correctly identified (Table 2). The mechanism of buprenorphine was the least commonly identified at 60% (*n* = 256), and naltrexone was correctly identified 64% (*n* = 272) of the time. Only 43% of participants correctly identified the mechanism of all 3 medications and 13% (*n* = 57) of participants did not identify the mechanism of any MOUD correctly.

**Table 2.** Percent correctly identifying each medication (*n* = 403).

	% of sample	All 3 correct	Bup correct	Methadone correct	Naltrexone correct
All	100%	43.3%	60.2%	67.1%	64.0%
Provider type					
Nurse practitioner	30.0%	34.7%**	53.7%*	59.5%*	59.5%
Physician associate	27.0%	41.3%	56.9%	63.3%	62.4%
Physician	43.1%	52.9%**	70.7%**	76.4%**	70.1%*
Specialty					
Family medicine	54.2%	40.4%	56.1%	66.1%	61.7%
Addiction medicine	10.7%	78.0%***	88.0%***	86.0%**	84.0%**
Emergency medicine	16.8%	36.8%	58.8%	55.9%	58.8%
Infectious disease	2.9%	46.2%	69.2%	61.5%	69.2%
Internal medicine	17.5%	48.7%	62.8%	73.1%	70.5%
Obstetrics/gynecology	4.6%	30.0%	65.0%	75.0%	51.3%
Pain medicine	1.0%	80.0%	80.0%	80.0%	80.0%
Psychiatry	5.5%	52.2%	65.2%	65.2%	78.3%
Training					
Previously X-waivered	48%	57%***	72%***	72%*	75%***
Practice location					
Rural	21%	45%	57%	65%	67%
Urban	79%	44%	63%	68%	64%

* *p* < 0.05, **p < 0.01, ***p < 0.001.

Of the three types of health care providers eligible to prescribe, NPs correctly identified the mechanism of each type of medication least often. Only 35% of NPs correctly identified all three medications, compared to 41% of PAs and 51% of physicians. In a chi-squared test, these differences were statistically significant (Χ^2^_(402)_=10.09, *p* < 0.01, φ_c_=0.16). Moreover, 54% of NPs correctly identified the mechanism of buprenorphine, compared to 57% of PAs and 71% of physicians (Χ^2^_(402)_=10.30, *p* < 0.01, φ_c_=0.16). Sixty percent of NPs identified the mechanism of methadone correctly, as compared to 63% of PAs and 76% of physicians (Χ^2^_(402)_=10.77, *p* < 0.01, φ_c_ =0.16). Finally, 47% of physicians identified the mechanism of naltrexone correctly, as compared to 27% of NPs and 26% of PAs, but these differences were not statistically significant (Χ^2^_(402)_=3.92, *p* = 0.14, φ_c_ = −0.10).

**Figure 1.**
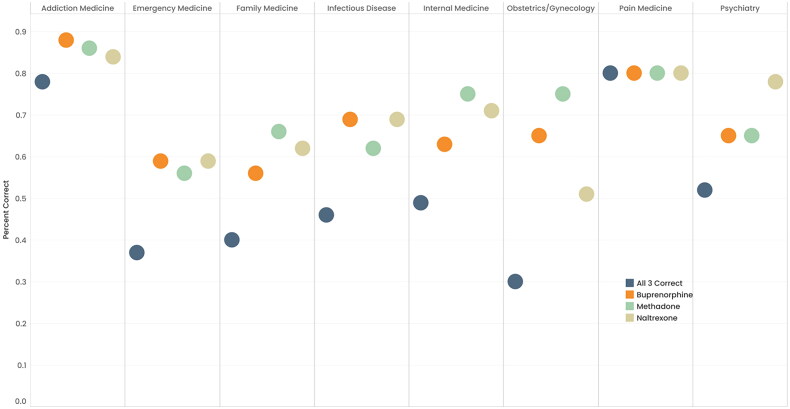
Percent of health-care professionals correctly identifying MOUD by specialty.

The percentage of participants correctly identifying the mechanism of MOUD also varied by training specialty (Figure 1). Pain management and addiction medicine providers were the most likely to correctly identify the mechanism of all three medications, at 80% (*n* = 5) and 78% (*n* = 56) of participants, respectively. Emergency Medicine and Obstetrics/Gynecology providers were the least likely to identify the mechanism of all three correctly, at 37% (*n* = 24) and 30% (*n* = 88), respectively. Participants who previously received an X-waiver were more likely to correctly identify all 3 medications (*p* < 0.01). 57% (*n* = 120) of participants with an X-waiver correctly identified all 3 medications versus 30% (*n* = 63) of participants without an X-waiver. No significant differences in knowledge emerged for participants practicing in rural versus urban counties in Ohio.

### Multivariable regression

After controlling for provider characteristics and empathy toward patients with OUD, less medication knowledge was associated with more negative attitudes towards patients with OUD and MOUD treatment (Table 3). Participants who correctly identified the mechanism of all three MOUD had significantly lower bias towards patients with OUD (*b* = −0.18, 95% CI: −0.30, −0.07). In addition, participants who correctly identified the mechanism of MOUD were significantly less likely to believe that opioid use was a choice or controllable (*b* = −0.14, 95% CI: −0.29, −0.01); and to believe they personally could not become addicted to opioids (*b* = −0.22, 95% CI: −0.39, −0.06). Finally, MOUD knowledge was associated with lower confidence in non-MOUD treatment strategies, including abstinence-only treatment programs and mutual support groups alone (*b* = −0.27, 95% CI: −0.48, −0.07).

**Table 3.** Multivariable regression results of the association between medication knowledge and treatment attitudes.

*n* = 403	Bias			Controllability			Vulnerability			Abstinence-based recovery		
	Coef.	SE	95% CI	Coef.	SE	95% CI	Coef.	SE	95% CI	Coef.	SE	95% CI
Unadjusted												
Knowledge	−0.29***	0.06	−0.38, −0.15	−0.21**	0.07	−0.35, −0.06	−0.30***	0.08	0–0.46, 0–0.13	−0.28**	0.10	−0.48, −0.08
Adjusted												
Knowledge	−0.18**	0.60	−0.30, −0.07	−0.14*	0.07	−0.29, −0.00	−0.22*	0.60	−0.30, −0.07	−0.27*	0.12	−0.50, −0.07
NP	0.27	0.77	−0.12, 0.18	0.16	0.09	−0.03, 0.34	0.14	0.77	−0.12, 0.18	0.11	0.14	−0.16, 0.40
PA	−0.029	0.77	−0.18, 0.12	0.11	0.09	−0.08, 0.29	−0.09	0.77	−0.18, 0.12	0.21	0.14	−0.06, 0.50
Female	−0.24***	0.66	−0.37, −0.11	−0.28**	0.08	−0.44, −0.12	−0.04	0.66	−0.37, −0.11	−0.25*	0.12	−0.50, −0.02
Age	−0.00	0.00	−0.01, 0.00	−0.01	0.00	−0.01, 0.00	−0.00	0.00	−0.01, 0.00	−0.01	0.00	−0.02, 0.00
Empathy	−0.38***	0.03	−0.44, −0.32	−0.20***	0.04	−0.27, −0.13	−0.16***	0.03	−0.44, −0.32	0.02	0.05	−0.09, 0.12
Hours/Week	−0.00	0.00	−0.01, 0.00	−0.00	0.00	−0.01, 0.00	−0.00	0.00	−0.01, 0.00	−0.00	0.00	−0.01, 0.01
Cons	3.9	0.22	3.47, 4.34	3.6	0.27	3.06, 4.14	4.17	0.32	3.54, 4.80	3.3	0.40	2.54, 4.10

* *p* < 0.05; **p < 0.01; ***p < 0.001.

## Discussion

We found considerable variation in knowledge of MOUD among currently active health care professionals in Ohio. Of the three medications used to treat OUD, participants least often identified the mechanism of action for buprenorphine correctly. This finding is important given efforts to expand buprenorphine access in outpatient treatment settings and particularly primary care [42–44]. Limited knowledge on buprenorphine may be an important barrier to overcome to increase adoption among HCPs. Overall, fewer than half of HCP participants could identify all three medications correctly. These findings suggest that existing training on MOUD prescribing is insufficient [17,45].

We also found significant variation in who held accurate knowledge of MOUD. When comparing all three types of eligible MOUD prescribers, NPs were the least likely to correctly identify each type of medication. NPs play a vital role in expanding MOUD access as they are a rapidly growing group of HCPs and often provide primary care in rural and underserved areas [46]. Although there are critical knowledge gaps for currently practicing HCPs of all provider types, there may be a unique opportunity to expand education on OUD for NPs. Future research should work to adapt training interventions to engage different types of MOUD prescribers.

Health-care professional knowledge of MOUD also varied by specialty. Family medicine providers were the least likely to identify the mechanism of buprenorphine correctly. This finding is significant, given the potential for buprenorphine prescribing in primary care to help address the profound MOUD access gap [47,48]. OB/GYN providers also less often identified all three medications correctly. Lack of knowledge among OB/GYN providers may limit evidence-based treatment for individuals with perinatal opioid use, a significant public health concern associated with adverse maternal and fetal outcomes [49].

Emergency medicine providers also had lower scores across all three medications, an important finding given that hospital visits are considered a critical window for initiating MOUD [50]. A number of evidence-based treatment models, often called transitional opioid programs, have been developed within emergency departments that aim to begin MOUD and link patients to ongoing treatment in the community [51], but limited MOUD knowledge among emergency medicine providers may limit the successful scale-up of these programs. Unsurprisingly, pain management and addiction medicine providers were the most likely to identify buprenorphine, methadone, and naltrexone correctly.

Importantly, lower knowledge related to MOUD among participants was also significantly and positively associated with a range of negative attitudes toward MOUD treatment and patients with OUD, even after controlling for provider characteristics and general empathy toward patients with OUD. HCPs with higher knowledge scores were less likely to view OUD as controllable or a result of poor decision-making; were less likely to hold stigmatizing views of patients with OUD; were more likely to believe that they could be vulnerable to addiction themselves; and were less likely to prefer OUD treatment that did not include medication.

Previous studies have demonstrated that improving knowledge can decrease stigma. One potential mechanism for this effect is that knowledge improvement reduces reliance on harmful stereotypes, and as a result, improves attitudes. In the context of OUD, improved knowledge about the mechanisms of MOUD could dispel stereotypes about individuals with OUD being responsible for their addiction and provide evidence that OUD is a chronic brain disease. Improved knowledge of MOUD mechanisms could also reduce stereotypes about medication treatment. For example, understanding the differences between the three types of MOUD and more familiar opioid agonists used to treat pain and disproportionately contributed to the opioid epidemic [52,53] could combat common misperceptions about MOUD. For example, persistent stereotypes include that MOUD just substitute one drug for another [16,54], are unsafe [16,55], and do not truly constitute recovery as MOUD treatment does not require abstaining from all substances [54]. Although our findings do not allow for causal interpretation, the association between knowledge and attitudes toward OUD and MOUD warrants future research to develop and test implementation strategies to improve both knowledge of and attitudes towards MOUD.

## Public health implications

These findings have implications for patients with OUD and their ability to access sufficient healthcare and treatment services. Our results suggest that for many HCPs, current training is not adequate to correctly identify the mechanism of MOUD. Given that improving knowledge is a critical step to increasing prescribing [17], these findings suggest that additional training is warranted not only in health professions educational settings, but across the career course. Importantly, participants with low knowledge of MOUD were also more likely to hold stigmatizing attitudes towards patients with OUD; were more likely to view addiction as a choice; and were more likely to prefer abstinence or mutual support groups for opioid use disorder treatment as compared to MOUD.

Importantly, existing studies provide some evidence that educational interventions are related to increased buprenorphine prescribing [56–58]. Our results suggest that one possible explanation for improvement is that increasing knowledge also reduces negative attitudes towards patients with OUD and toward MOUD. Because our data are cross-sectional, it is not possible to say whether improved training led to less stigmatizing attitudes, or whether individuals with more positive attitudes toward OUD and MOUD were more likely to pursue additional training. Regardless, future training interventions should include both knowledge-based training to increase confidence with using different MOUD and content to reduce stigma, such as facilitating contact with patients with opioid use disorder given the evidence that both are important for improving willingness to prescribe buprenorphine [17]. Our findings build on previous literature as it demonstrates the need to further equip HCPs with the knowledge and skills to effectively work with and treat patients with OUD [59]. Findings from the current study can also aid in those efforts by identifying the specific subgroups of HCPs who might benefit the most from tailored training or other interventions.

## Limitations

Although we drew from a larger sample (*n* = 403) than what was used in previous studies, our participants were all HCPs from Ohio, which limits generalizability to other regions, especially because Ohio has been disproportionately affected by opioid overdoses [60,61]. Additionally, we collected survey responses via an anonymous, online questionnaire. Although this approach is convenient and can help limit social desirability bias, we did not use a probability sampling approach and our sample could have included participants who have stronger views on treating opioid use disorder and were thus motivated to take the survey. Further, participants from some specialties were underrepresented in our sample, particularly pain management and infectious disease which is important given the high level of contact between these HCPs and patients with OUD [62,63]. Finally, although providers were compensated $20 for participation in our study, it may have been too low to incentivize them, given their median salaries.

## Conclusion

Ensuring access to MOUD treatment is critically important and requires a trained and willing HCP workforce to prescribe these medications. Our findings highlight considerable variation in clinician knowledge of MOUD and suggest that knowledge levels are also related to negative attitudes towards patients with OUD and MOUD. Future training interventions should be tailored to learners in different settings (health professions education and providers already in practice), as well as to advanced practice providers who make up a large part of the workforce in certain regions of the United States. Training interventions that increase knowledge as well as focus on stigma reduction are critical for reducing the longstanding treatment gap for opioid use disorder.

## Data Availability

The study data will be made available by the authors upon reasonable request.

## References

[1] Garcia S, Teater J, Trimble C, et al. Years of life lost due to unintentional drug overdose relative to the leading underlying causes of death in the United States: a comparative analysis of excess mortality 2017-2019. J Addict Dis. 2024;42(3):205–209. doi: 10.1080/10550887.2023.2173929. 36876385

[2] CDC WONDER. Accessed August 23, 2023. https://wonder.cdc.gov/.

[3] Drug overdose deaths – Health, United States; 2023. Accessed September 14. https://www.cdc.gov/nchs/hus/topics/drug-overdose-deaths.htm.

[4] SAMHSA applauds expansion of access to medication for opioid use disorder (MOUD); 2023. SAMHSA. Accessed August 23. https://www.samhsa.gov/newsroom/statements/2023/samhsa-applauds-expansion-access-moud.

[5] Madras BK, Ahmad NJ, Wen J, et al. Improving access to evidence-based medical treatment for opioid use disorder: strategies to address key barriers within the treatment system. NAM Perspect. 2020;2020. doi: 10.31478/202004B. PMC8916813 35291732

[6] Waller RC, Clark KJ, Woodruff A, et al. Guide for future directions for the addiction and OUD treatment ecosystem. NAM Perspect. 2021;2021. doi: 10.3147/202104B. PMC8406500 34532686

[7] Center for Behavioral Health Statistics S. Key substance use and mental health indicators in the United States: results from the 2020 National Survey on Drug Use and Health. Subst Abuse: Res Treat; 2021.

[8] Dickson-Gomez J, Spector A, Weeks M, et al. “You’re Not Supposed to be on it Forever”: medications to treat opioid use disorder (MOUD) related stigma among drug treatment providers and people who use opioids. Subst Abuse. 2022;16:11782218221103859. doi: 10.1177/11782218221103859. 35783464 PMC9243471

[9] Mackey K, Veazie S, Anderson J, et al. Barriers and facilitators to the use of medications for opioid use disorder: a rapid review. J Gen Intern Med. 2020;35(Suppl 3):954–963. doi: 10.1007/S11606-020-06257-4. 33145687 PMC7728943

[10] Carl A, Pasman E, Broman MJ, et al. Experiences of healthcare and substance use treatment provider-based stigma among patients receiving methadone. Drug Alcohol Depend Rep. 2023;6:100138. doi: 10.1016/J.DADR.2023.100138. 36994374 PMC10040326

[11] Austin EJ, Briggs ES, Ferro L, et al. Integrating routine screening for opioid use disorder into primary care settings: experiences from a National Cohort of Clinics. J Gen Intern Med. 2023;38(2):332–340. doi: 10.1007/S11606-022-07675-2. 35614169 PMC9132563

[12] Klusaritz H, Bilger A, Paterson E, et al. Impact of stigma on clinician training for opioid use disorder care: a qualitative study in a primary care learning collaborative. Ann Fam Med. 2023;21(Suppl 2):S31–S38. doi: 10.1370/AFM.2920. 36849482 PMC9970664

[13] Oros SM, Christon LM, Barth KS, et al. Facilitators and barriers to utilization of medications for opioid use disorder in primary care in South Carolina. Int J Psychiatry Med. 2021;56(1):14–39. doi: 10.1177/0091217420946240. 32726568 PMC10954352

[14] Lundin RM, Hill H. The worrying, current state of addictions training in medicine. Front Med (Lausanne). 2022;9:1062096. doi: 10.3389/FMED.2022.1062096. 36507515 PMC9731765

[15] Rasyidi E, Wilkins JN, Danovitch I. Training the next generation of providers in addiction medicine. Psychiatr Clin North Am. 2012;35(2):461–480. doi: 10.1016/J.PSC.2012.04.001. 22640766

[16] Garett R, Young SD. The role of misinformation and stigma in opioid use disorder treatment uptake. Subst Use Misuse. 2022;57(8):1332–1336. doi: 10.1080/10826084.2022.2079133. 35611913 PMC9773633

[17] Jakubowski A, Singh-Tan S, Torres-Lockhart K, et al. Hospital-based clinicians lack knowledge and comfort in initiating medications for opioid use disorder: opportunities for training innovation. Addict Sci Clin Pract. 2023;18(1):31. doi: 10.1186/S13722-023-00386-X/TABLES/3. 37198707 PMC10193697

[18] Paul N, Kennedy AJ, Taubenberger S, et al. Provider perceptions of medication for opioid used disorder (MOUD): a qualitative study in communities with high opioid overdose death rates. Subst Abus. 2022;43(1):742–748. doi: 10.1080/08897077.2021.2007518. 35100094 PMC10960355

[19] Morgan AJ, Reavley NJ, Ross A, et al. Interventions to reduce stigma towards people with severe mental illness: systematic review and meta-analysis. J Psychiatr Res. 2018;103:120–133. doi: 10.1016/J.JPSYCHIRES.2018.05.017. 29843003

[20] Waqas A, Malik S, Fida A, et al. Interventions to reduce stigma related to mental illnesses in educational institutes: a systematic review. Psychiatr Q. 2020;91(3):887–903. doi: 10.1007/S11126-020-09751-4/FIGURES/5. 32372401 PMC7395002

[21] Gronholm PC, Henderson C, Deb T, et al. Interventions to reduce discrimination and stigma: the state of the art. Soc Psychiatry Psychiatr Epidemiol. 2017;52(3):249–258. doi: 10.1007/S00127-017-1341-9/TABLES/2. 28144713 PMC5344948

[22] Livingston JD, Milne T, Fang ML, et al. The effectiveness of interventions for reducing stigma related to substance use disorders: a systematic review. Addiction. 2012;107(1):39–50. doi: 10.1111/J.1360-0443.2011.03601.X. PMC3272222 21815959

[23] Bielenberg J, Swisher G, Lembke A, et al. A systematic review of stigma interventions for providers who treat patients with substance use disorders. J Subst Abuse Treat. 2021;131:108486. doi: 10.1016/J.JSAT.2021.108486. 34217033

[24] Corrigan PW, Penn DL. Lessons from social psychology on discrediting psychiatric stigma. Am Psychol. 1999;54(9):765–776. doi: 10.1037/0003-066X.54.9.765. 10510666

[25] Corrigan PW, River PL, Lundin RK, et al. Three strategies for changing attributions about severe mental illness. Schizophr Bull. 2001;27(2):187–195. doi: 10.1093/OXFORDJOURNALS.SCHBUL.A006865. 11354586

[26] Morgan AJ, Ross A, Reavley NJ. Systematic review and meta-analysis of Mental Health First Aid training: effects on knowledge, stigma, and helping behaviour. PLoS One. 2018;13(5):e0197102. doi: 10.1371/JOURNAL.PONE.0197102. 29851974 PMC5979014

[27] Volkow ND, Koob GF, McLellan AT. Neurobiologic advances from the brain disease model of addiction. N Engl J Med. 2016;374(4):363–371. doi: 10.1056/NEJMRA1511480. 26816013 PMC6135257

[28] Hall W, Carter A, Forlini C. The brain disease model of addiction: is it supported by the evidence and has it delivered on its promises? Lancet Psychiatry. 2015;2(1):105–110. doi: 10.1016/S2215-0366(14)00126-6. 26359616

[29] Meurk C, Carter A, Partridge B, et al. How is acceptance of the brain disease model of addiction related to Australians’ attitudes towards addicted individuals and treatments for addiction? BMC Psychiatry. 2014;14(1):373. doi: 10.1186/S12888-014-0373-X/TABLES/7. 25539621 PMC4297372

[30] Hammer R, Dingel M, Ostergren J, et al. Addiction: current criticism of the brain disease paradigm. AJOB Neurosci. 2013;4(3):27–32. doi: 10.1080/21507740.2013.796328. 24693488 PMC3969751

[31] Kelly JF, Greene MC, Abry A. A US national randomized study to guide how best to reduce stigma when describing drug‐related impairment in practice and policy. Addiction. 2021;116(7):1757–1767. doi: 10.1111/ADD.15333. 33197084 PMC8124079

[32] Van Boekel LC, Brouwers EPM, Van Weeghel J, et al. Stigma among health professionals towards patients with substance use disorders and its consequences for healthcare delivery: systematic review. Drug Alcohol Depend. 2013;131(1-2):23–35. doi: 10.1016/J.DRUGALCDEP.2013.02.018. 23490450

[33] Kiang MV, Basu S, Chen J, et al. Assessment of changes in the geographical distribution of opioid-related mortality across the United States by opioid type, 1999-2016. JAMA Netw Open. 2019;2(2):e190040. doi: 10.1001/JAMANETWORKOPEN.2019.0040. 30794299 PMC6484620

[34] Rule 4731-33-03 – Ohio Administrative Code. Ohio Laws. Accessed January 29, 2024. https://codes.ohio.gov/ohio-administrative-code/rule-4731-33-03.

[35] Franz B, Dhanani LY, Miller WC. Rural-urban differences in physician bias toward patients with opioid use disorder. Psychiatr Serv. 2021;72(8):874–879. doi: 10.1176/appi.ps.202000529. 33622043

[36] Franz B, Dhanani LY, Brook DL. Physician blame and vulnerability: novel predictors of physician willingness to work with patients who misuse opioids. Addict Sci Clin Pract. 2021;16(1):33. doi: 10.1186/S13722-021-00242-W. 34034825 PMC8147073

[37] Brener L, Von Hippel W. Measuring attitudes toward injecting drug users and people with hepatitis C. Subst Use Misuse. 2008;43(3-4):295–302. doi: 10.1080/10826080701202627. 18365932

[38] Dhanani LY, Miller WC, Hall OT, et al. Positive contact and empathy as predictors of primary care providers’ willingness to prescribe medications for opioid use disorder. SSM – Mental Health. 2023;4:100263. doi: 10.1016/j.ssmmh.2023.100263. 38558957 PMC10979326

[39] Ohio Rural Health Improvement Plan. Ohio Rural Health Association. Accessed February 26, 2024. https://www.ohioruralhealth.org/ohio-rural-health-improvement-plan.

[40] Gelman A, Hill J, Vehtari A. Regression and other stories. Regression and Other Stories. 2020. doi: 10.1017/9781139161879.

[41] Stata. FAQ. Citing Stata software, documentation, and FAQs. Accessed September 14, 2023. https://www.stata.com/support/faqs/resources/citing-software-documentation-faqs/.

[42] Substance Abuse and Mental Health Services Administration (SAMHSA). Practical tools for prescribing and promoting buprenorphine in primary care settings. 2021.

[43] Nakaishi L, Sugden SG, Merlo G. Primary care at the intersection of lifestyle interventions and unhealthy substance use. Am J Lifestyle Med. 2023;17(4):494–501. doi: 10.1177/15598276221111047. 37426739 PMC10328212

[44] Cloutier RM, Cole ES, McDonough BL, et al. Strategies to recruit rural primary care providers to implement a medication for opioid use disorder (MOUD) focused integrated care model. Implement Res Pract. 2023;4:26334895231152808. doi: 10.1177/26334895231152808/FORMAT/EPUB. 37091535 PMC9978659

[45] Haffajee RL, Bohnert ASB, Lagisetty PA. Policy pathways to address provider workforce barriers to buprenorphine treatment. Am J Prev Med. 2018;54(6 Suppl 3):S230–S242. doi: 10.1016/J.AMEPRE.2017.12.022. 29779547 PMC6330240

[46] Banka-Cullen SP, Comiskey C, Kelly P, et al. Nurse prescribing practices across the globe for medication-assisted treatment of the opioid use disorder (MOUD): a scoping review. Harm Reduct J. 2023;20(1):78. doi: 10.1186/S12954-023-00812-Y/TABLES/3. 37353762 PMC10288784

[47] Brackett CD, Duncan M, Wagner JF, et al. Multidisciplinary treatment of opioid use disorder in primary care using the collaborative care model. Subst Abus. 2022;43(1):240–244. doi: 10.1080/08897077.2021.1932698. 34086531

[48] Tong S, Sabo R, Aycock R, et al. Assessment of addiction medicine training in family medicine residency programs: a CERA study. Fam Med. 2017;49(7):537–543. Accessed August 23, 2023. /pmc/articles/PMC6996005/28724151 PMC6996005

[49] Tobon AL, Habecker E, Forray A. Opioid use in pregnancy. Curr Psychiatry Rep. 2019;21(12):118. doi: 10.1007/S11920-019-1110-4. 31734808 PMC10296780

[50] Franz B, Cronin CE, Pagan JA. What strategies are hospitals adopting to address the opioid epidemic? Evidence from a national sample of nonprofit hospitals. Public Health Rep. 2021;136(2):228–238. doi: 10.1177/0033354920968805. 33176117 PMC8093846

[51] Shanahan CW, Beers D, Alford DP, et al. A transitional opioid program to engage hospitalized drug users. J Gen Intern Med. 2010;25(8):803–808. doi: 10.1007/S11606-010-1311-3. 20237960 PMC2896583

[52] Madden EF, Barker KK, Guerra J, et al. Variation in intervention stigma among medications for opioid use disorder. *SSM – Qualitative Research in Health*. 2022;2:100161. doi: 10.1016/j.ssmqr.2022.100161.

[53] Haffajee RL, Mello MM. Drug companies’ liability for the opioid epidemic. N Engl J Med. 2017;377(24):2301–2305. doi: 10.1056/NEJMP1710756. 29236640 PMC7479783

[54] Madden EF, Prevedel S, Light T, et al. Intervention stigma toward medications for opioid use disorder: a systematic review. Subst Use Misuse. 2021;56(14):2181–2201. doi: 10.1080/10826084.2021.1975749. 34538213

[55] Velander JR. Suboxone: rationale, science, misconceptions. Ochsner J. 2018;18(1):23–29. doi: 10.1043/TOJ-17-0095. 29559865 PMC5855417

[56] Chen L, Sethi S, Poland C, et al. Prescriptions for buprenorphine in Michigan following an education intervention. JAMA Netw Open. 2023;6(12):e2349103-e2349103. doi: 10.1001/JAMANETWORKOPEN.2023.49103. 38127344 PMC10739087

[57] Clark B, Kai M, Dix R, et al. Association of a multimodal educational intervention for primary care physicians with prescriptions of buprenorphine for opioid use disorders. JAMA Netw Open. 2019;2(10):e1913818-e1913818. doi: 10.1001/JAMANETWORKOPEN.2019.13818. 31642929 PMC6820030

[58] Khatri UG, Lee K, Lin T, et al. A brief educational intervention to increase ED initiation of buprenorphine for opioid use disorder (OUD). J Med Toxicol. 2022;18(3):205–213. doi: 10.1007/S13181-022-00890-7/FIGURES/3. 35415804 PMC9004452

[59] Dhanani LY, Harris EL, Mirto J, et al. Barriers to working with patients who misuse opioids and physician burnout: implications for medical education. Subst Use Misuse. 2022;57(8):1177–1184. doi: 10.1080/10826084.2022.2069264. 35473470

[60] Hall OT, Hall OE, Kolodny A, et al. Assessment of excess mortality associated with drug overdose in Ohio from 2009 to 2018. JAMA Netw Open. 2020;3(4):e202183-e202183. doi: 10.1001/JAMANETWORKOPEN.2020.2183. 32259262 PMC7139273

[61] Hall OT, Hall OE, McGrath RP, et al. Years of life lost due to opioid overdose in Ohio: temporal and geographic patterns of excess mortality. J Addict Med. 2020;14(2):156–162. doi: 10.1097/ADM.0000000000000554. 31592905

[62] Latif Z-EH, Skjaervø I, Solli KK, et al. Chronic pain among patients with an opioid use disorder. Am J Addict. 2021;30(4):366–375. doi: 10.1111/AJAD.13153. 33738870

[63] Rosenblatt RA, Andrilla CHA, Catlin M, et al. Geographic and specialty distribution of US physicians trained to treat opioid use disorder. Ann Fam Med. 2015;13(1):23–26. doi: 10.1370/AFM.1735. 25583888 PMC4291261

